# ACPSEM position paper: the safety of magnetic resonance imaging linear accelerators

**DOI:** 10.1007/s13246-023-01224-9

**Published:** 2023-02-27

**Authors:** Nick Cook, Nikki Shelton, Stephen Gibson, Peter Barnes, Reza Alinaghi-Zadeh, Michael G. Jameson, on behalf of the ACPSEM Magnetic Resonance Imaging Linac Working Group (MRILWG)

**Affiliations:** 1 Christchurch Hospital, Christchurch, New Zealand; 2 Olivia Newton-John Cancer Wellness and Research Centre, Heidelberg, VIC Australia; 3 Townsville Cancer Centre, Douglas, QLD Australia; 4 Austin Health, Heidelberg, VIC Australia; 5 GenesisCare, Sydney, NSW Australia; 6 University of New South Wales, Sydney, Australia

**Keywords:** Magnetic resonance imaging, Linear accelerator, Adaptive radiation therapy, Safety, Training, Guidance

## Abstract

Magnetic Resonance Imaging linear-accelerator (MRI-linac) equipment has recently been introduced to multiple centres in Australia and New Zealand. MRI equipment creates hazards for staff, patients and others in the MR environment; these hazards must be well understood, and risks managed by a system of environmental controls, written procedures and a trained workforce. While MRI-linac hazards are similar to the diagnostic paradigm, the equipment, workforce and environment are sufficiently different that additional safety guidance is warranted. In 2019 the Australasian College of Physical Scientists and Engineers in Medicine (ACPSEM) formed the Magnetic Resonance Imaging Linear-Accelerator Working Group (MRILWG) to support the safe clinical introduction and optimal use of MR-guided radiation therapy treatment units. This Position Paper is intended to provide safety guidance and education for Medical Physicists and others planning for and working with MRI-linac technology. This document summarises MRI-linac hazards and describes particular effects which arise from the combination of strong magnetic fields with an external radiation treatment beam. This document also provides guidance on safety governance and training, and recommends a system of hazard management tailored to the MRI-linac environment, ancillary equipment, and workforce.

## Introduction

### Background

The safe clinical introduction and optimal use of MRI-linacs is a multidisciplinary challenge requiring input from and collaboration between many professions, hospital departments, and external suppliers. ACPSEM seeks to meet this challenge by influencing the quality and delivery of MRI-linac therapies. ACPSEM aims to prepare the Medical Physics workforce to do this safely by providing leadership through this position paper and the ACPSEM Magnetic Resonance Safety Expert Certification course.

Guidance on safety issues in diagnostic MRI is provided by RANZCR MRI Safety Guidelines [1]. All guidance given herein is in addition to and is intended to be consistent with existing RANZCR recommendations, which should also be followed. Further MR safety information is also available from the American College of Radiology (ACR) [2] and the Medicines and Healthcare products Regulatory Agency (MHRA) [3]. This ACPSEM guidance considers a first MRI-linac installation with an inexperienced MR workforce where education and diagnostic support is emphasised; as experience is gained this support becomes less important, but ongoing collaboration is strongly encouraged.

### Position paper scope

This position paper is intended as a guidance document and educational resource to assist medical physicists in the safe use of MRI-linac technology. The paper will:

- recommend a consensus safety governance structure [4] with appropriate personnel to fit key roles within that structure
- introduce the main hazards associated with MRI-linac technology and practice
- discuss MRI-linac site planning and management issues with recommended safety zones and access controls
- recommend staff training levels and responsibilities with regard to safety including screening
- discuss safe ancillary equipment management, MR safety labelling, and in-house safety assessments
- discuss patient management issues including implants and interpretation of manufacturer’s MR conditions

Specific hazards are mentioned throughout the position paper with recommended risk reduction and management strategies. Recommendations are made recognising that there may be equally valid alternative solutions for reducing specific risks. MRI-Simulation scanners are not specifically included in the scope though the issues discussed, and guidance presented may still be applicable. Any mention of particular technologies or models does not imply endorsement by ACPSEM.

### MRI guided radiotherapy (MRIgRT)

**Fig. 1:**
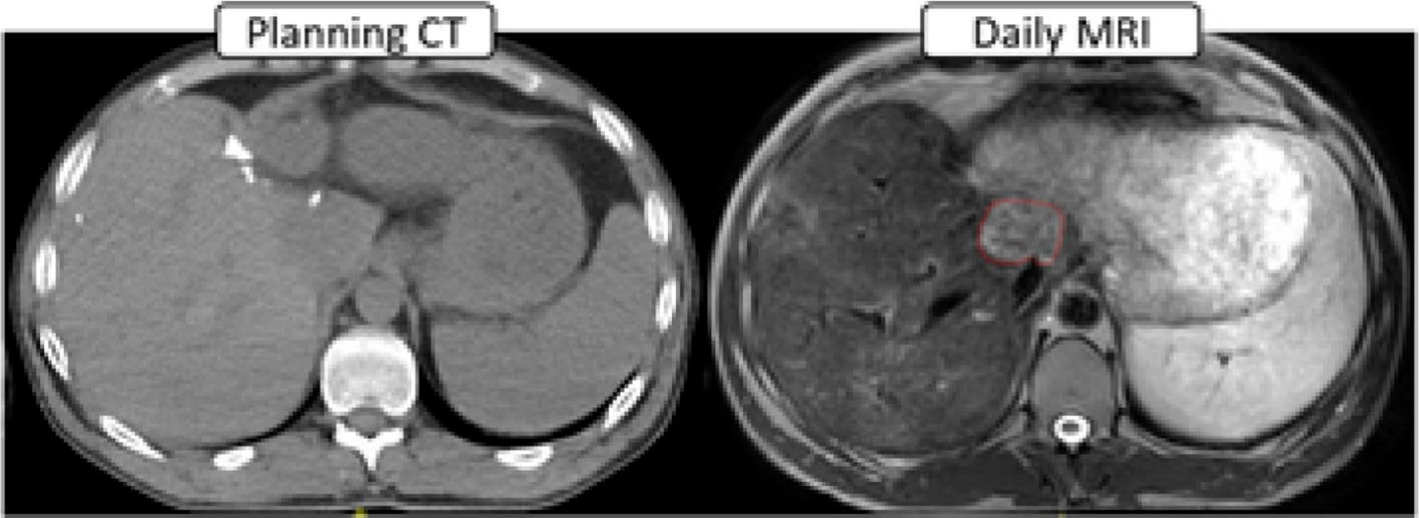
Abnormal tissue (circled on right) is more clearly visualised and defined on MRI than CT

The combination of an MRI scanner and radiation therapy treatment machine is a recent technological innovation [5]. It enables daily monitoring of, and treatment adaption [6] to, anatomical and physiological changes in tumour position, size and shape as well as the possibility of real-time tracking of tumour position with respiratory motion (or other motion inducing anatomical changes). One of the main drivers for MRI-linac development is to bring MR imaging into the treatment room to access the superior soft tissue visualisation (Fig. 1) compared to cone beam computed tomography (CBCT). However, MR imaging units have only recently been introduced to many radiotherapy departments; this represents a risk where staff may not have appropriate training and/or experience working with strong magnetic fields.

### Current MRI-linear accelerators

**Fig. 2:**
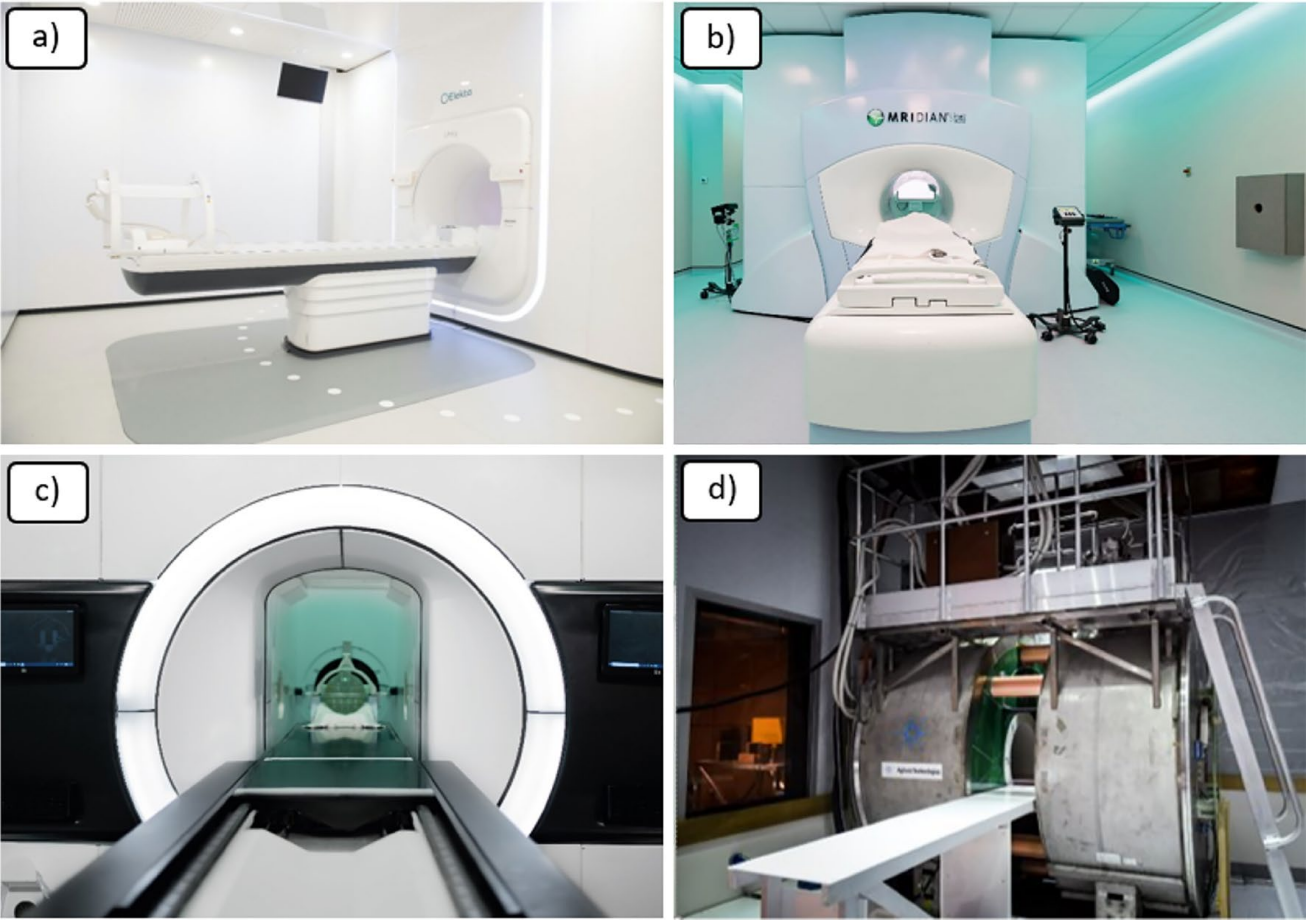
Current MRI-linac systems **a** Elekta Unity, **b** ViewRay MRIdian, **c** MagnetTx Aurora and **d** Australian MRI-linac

There are four MRI-linacs in development globally [7], these are shown in Fig. 2. Two are commercially available clinical systems, the Elekta Unity [8] (Elekta Solutions AB, Stockholm, Sweden) and ViewRay MRIdian [9] (Viewray Technologies Inc, Cleveland, OH, USA). There are also two research systems, the MagnetTx Aurora [10] and the Australian MR-linac [11].

The characteristics of the systems are detailed in Table 1. Both clinical systems have 6–7 MV flattening filter free (FFF) beams with 0.35 and 1.5 T magnets for the MRIdian and Unity respectively. The source image distance (SID) varies from 0.9 to 1.47 m and both are perpendicular in design. Beam orientation is described depending on whether the radiation beam is aligned parallel or perpendicular to the magnetic field. The research systems vary in field strength from 0.5 to 1.0 T and beam energy from 4 to 6 MV. Although the field strength of some MRI-linac systems is in line with standard diagnostic systems the gradient coils and other components may be different.

**Table 1:** Characteristics of current MRI-linac systems (modified from Whelan et al. [7])

System	Radiation type	Field strength (T)	Magnet type	Beam Orientation	SID (m)
Elekta unity	7 MV FFF	1.5	Closed superconducting	Perpendicular	1.435
ViewRay MRIdian	6 MV FFF	0.35	Split superconducting	Perpendicular	0.9
MagnetTx Aurora	6 MV	0.5	High temperature superconducting with steel yoke	Parallel	1.22
Australian MRI-linac	4 & 6 MV FFF	1.0	Open superconducting	Parallel with perpendicular option	1.9–3.3

### MRI-linac safety considerations

The MR environment is the source of many varied and often non-intuitive hazards, some of which may be life threatening but can be managed with appropriate protocols and a well-trained workforce. Safety should be considered as early as possible and throughout the planning, commissioning and clinical operation of the MRI-linac.

Appropriate assessment and management of risk requires specific expertise and training. A high proportion of MRI-linac patients have metallic implants, and treatment often requires the use of positioning equipment in the bore. Quality Assurance processes are frequent, require multiple devices, and can involve many people or one. Some devices may not have appropriate MR safety labelling so access to expertise in safety screening and hazard assessment is essential for patient and staff safety.

Adoption or adaption of diagnostic MR safety protocols and close collaboration with experienced diagnostic MRI staff is strongly recommended where possible. Significant contact hours for key staff such as Radiation Oncologists (ROs), senior Radiation Therapists (RTs) and Medical Physicists provides essential safety experience and access to resources, such contact should commence well before commissioning of a first MRI-linac.

MRI-linac technology is readily available but is at an early stage of development and may be subject to technological changes which could modify existing hazards or introduce new ones. The physics and safety principles described in this document will likely still be relevant to altered or novel situations but will need expert consideration.

## Safety governance

The legally responsible management or clinical representative should take responsibility for MRI-linac safety and establish a management system to enhance and promote safety. Following international consensus guidance [4], ACPSEM strongly recommends that three professional roles are established to manage the safe delivery of MRI-linac services. Appropriately qualified, registered, and experienced people should be appointed to these roles, specific tasks should be delegated, and expert consultation should be sought as necessary, especially when installing an MRI-linac for the first time with an inexperienced workforce.

### Established MRI-linac safety roles

#### Designated responsible person

Responsibility for the safe operation of the MRI-linac site must be explicitly assigned to a nominated medical practitioner, typically titled the MR Medical Director (MRMD) [1]. This role is significantly concerned with patient care in a clinical setting and is expected to be taken on by a Radiation Oncologist (RO) or the local MRI Radiologist responsible for diagnostic MRI safety. The MRMD may be called upon to assess the balance of risk and benefit for unusual scanning or treatment situations and must have a good understanding of the safety issues and all systems of work in the MRI-linac facility.

The MRMD will require sufficient training to understand the workflows and hazards of MRI-linac treatments and take responsibility for formulation and application of policies and procedures. A fuller description of the MRMD role and responsibilities is given in international consensus guidance [4].

#### MR safety officer (MRSO)

The MR safety officer is responsible for the day-to-day implementation of the site’s MRI-linac safety policies and procedures. The MRSO role would usually be filled by a senior radiation therapist (RT), an experienced diagnostic MRI technologist, or a radiation oncology medical physicist (ROMP) but others with suitable MR safety expertise could be appointed. multiple MRSOs could be appointed and a combination of RT and ROMP appointees could have advantages. If the MR Safety Expert (MRSE, described below) is not on-site, this role takes on more responsibility and one of the MRSO appointees should be a ROMP. The MRSO role should not usually be combined with the MRSE role unless staff numbers make this unavoidable such as at small centres.

MRSO responsibilities are described in consensus guidance [4] and include but are not limited to:

- To be readily available to operators of the MRI-linac at all times that the MRI-linac facility is accessible.
- Ensuring that proper policies and procedures for day-to-day MR safety are enforced.
- Developing, documenting, and introducing, in conjunction with the MRSE and under the authority of the MRMD, safe working procedures for the MRI-linac environment.
- Ensuring that adequate written safety procedures, work instructions, emergency procedures, and operating instructions are issued to all concerned after full consultation with the MRMD and the MRSE.
- Ensuring that appropriate measures for minimizing risks to health that arise from the use of or exposure to the MRI-linac equipment, as per the direction of the MRMD, are implemented and monitored.
- Managing hazards posed by the MRI-linac equipment, and monitoring the measures taken to protect against such hazards.

Prior to MRI-linac installation the MRSO must have significant educational opportunities and contact time with diagnostic MRI colleagues. Training should include screening and scanning observation plus in-depth MR safety training and a good understanding of safety resources. Local legislation may also include academic and clinical requirements for registration as an MR technologist.

#### MR safety expert (MRSE)

ACPSEM strongly recommends that all MRI-linac sites have access to an ACPSEM certified MR safety expert, who should ideally be in-house but could be external. The MRSE role would usually be filled by a medical physicist but others with MRSE certification and suitable MRI-linac safety expertise could be appointed. The role may also be filled by an experienced MRI-linac medical physicist from another facility. Day-to-day implementation of safety policy and procedures remains the responsibility of the MRSO.

The MRSE should have a deep technical knowledge of the MRI-linac equipment, physics and biophysics of electromagnetism, including interactions with implanted medical devices.

MRSE responsibilities are described in consensus guidance [4] and include but are not limited to:

- Providing safety advice regarding the selection, procurement, and installation of the MRI-linac system and related equipment, as well as on the quality control programs regarding their performance.
- Providing high-level advice on the engineering, scientific, and administrative aspects of the safe use of MR equipment.
- Providing advice on the development and continuing evaluation of a safety framework for the MR environment.
- Providing technical advice to assist the MRSO in developing safe working procedures in the MRI-linac environment, for approval by the MRMD.
- Provide safety advice regarding nonroutine MR procedures for individual patients and specific patient groups. e.g. patients with complex or novel implants.
- Liaise with neighbouring facilities to assess and manage impact of MRI-linac and potential quench on safety and correct function of devices.

### MR safety resources

All MRI-linac staff must have access to a wide range of safety information pertaining to risks likely to be encountered at the site. This information should be managed by the MRSO or MRSE and, include:

- Implanted devices and foreign bodies
- Patient positioning devices
- Quality assurance (QA) and ancillary equipment

Familiarity with diagnostic resources is a useful starting point and can be built on by adding MRI-linac-specific information as experience and knowledge is gained. This process should start well before MRI-linac installation so key staff are thoroughly familiar with hazards and procedures during commissioning.

The facility must maintain all necessary safety information pertaining to the function of the MRI-linac. This should include any training information provided by the MRI-linac supplier.

#### MR safety education

The MRSE should deliver or ensure the provision of annual refresher presentations on advanced MR safety topics to ROs, RTs, and Medical Physicists who work routinely in Zones III or IV.

For MRI-linac, refresher safety topics would include those listed in RANZCR Guidelines [1]:

- Specific absorption rate (SAR) and SAR control
- Peripheral nerve stimulation
- Device/implant conditionality and required procedures for assessing this
- Reading spatial gradient maps
- Understanding fringe field strengths and boundaries
- Emergency procedures in the case of patient cardiac arrest or equipment failure
- Emergency procedures in the event of a quench
- Safe monitoring technique of the Zone IV region

Plus, a special focus on the following:

- Patient positioning equipment
- QA and ancillary equipment
- Impact of magnetic field on the treatment beam

There should also be annual education sessions for visitors or staff from other departments who occasionally interact with the MRI-linac environment (e.g. cleaning, maintenance, and emergency personnel) explaining the MRI-linac environmental Zones, basic MR safety principles, and procedures for gaining access to appropriate parts of the MRI-linac suite.

### MR safety committee

An MR Safety committee should be established, chaired by the MRMD and comprise at least the MRSO, and the MRSE. The Committee should be formed at the planning stage and then monitor each stage of planning, commissioning and bringing the MRI-linac to service. For a first MRI-linac installation, it is highly recommended that experienced diagnostic MRI staff are either included in the MR safety committee or consulted in the adoption or formulation of policy and procedures. If circumstances allow, a joint diagnostic-therapeutic MR safety committee would be helpful in developing consistent policies and sharing resources and expertise.

The MR safety committee should meet at least annually to monitor establishment of policies and procedures, initiate reviews and audits, and manage any incident reports. Responsibility for formulation and application of policy remains with the MRMD.

### Documentation

There must be a safe practice manual paying particular attention to emergency situations as described in RANZCR Guidelines [1], including:

- Cardiac or respiratory arrest
- Contrast reaction
- Fire and quench

The manual should describe all hazards and include safe practices for all aspects of MRI-linac operation including:

- Scanning
- Planning
- Treatment
- Patient dosimetry
- Machine QA
- Ancillary equipment management
- Environmental monitoring

The safe practice manual should be reviewed by the MRSE at least annually, and with every hardware and major software modification.

An equipment register should be established with procedures for assessing, labelling and documenting the MR safety status of ancillary equipment, including patient positioning devices. Environmental monitoring should be established to capture any changes to structure, use, staff or equipment in the MRI-linac suite or in adjacent areas.

There must also be a documented incident reporting system, managed by the MRSO, audited by the MRSE, and reviewed by the MR Safety Committee. All adverse incidents must be reported to the MRMD.

The facility must maintain annual MR safety screening [1] information for all MRI-linac and other staff who enter MR Zones III and IV. All staff must immediately notify the MRSO of any change in their circumstances which could place them at increased risk in the MRI-linac environment, including any implanted or ferromagnetic device or object.

### Records of scans, plans and treatments

- A record of all scans, plans and treatments performed on each MRI-linac system should be maintained with key technical parameters. Electronic records must be appropriately backed up and accessible following any software or hardware change.
- As described by RANZCR guidelines [1], full details of any administered contrast agents should be recorded.

### Internal review and audit

There must be a program of regular review and audit of the following activities and policies:

- Quality assurance
- Screening
- Safe practice manual and other related documentation
- Incident reporting

A list of suggested audit topics is given in the Appendix. Such a list can never be exhaustive, and audits should always be tailored for the local site, equipment types, and procedures to regularly cover all aspects of MRI-linac safety. Audits and review should be controlled by the MRMD and MR safety committee.

### Relationship with stakeholders

The MR safety committee should encourage the formation of a multidisciplinary working group at an early stage of MRI-linac planning. All clinical and facility stakeholders should be identified and represented with oversight from senior clinicians and managers. MRMD, MRSO and MRSE participation should be from the outset and mandatory.

The MR safety committee should oversee a documented review of the plan of works for each stage of installation, commissioning and servicing, ensuring hazards are identified and managed appropriately. Safety protocols should be formally agreed with all organisations involved, this may include the MRI-linac supplier, commissioning and servicing agents, other departments, or other nearby facilities. The MRMD remains responsible for MRI-linac safety and must ensure that appropriate policies, procedures, and training are in place. During installation and commissioning of the MRI-linac and ancillary equipment, the MRMD may not have complete control over the MRI-linac environment or workers and to some extent may rely on the equipment vendor and installers to ensure the safety of their staff and any visitors. It is important that demarcations in space and time are agreed and established to ensure safe control and handover. Additional and temporary access controls and signage may be useful in safely managing the environment.

## MRI-linac parameters, fields, effects and hazards

The following sub-sections are intended to be an introduction to the physics, physiology, and hazards of MRI-linac equipment and to act as an education resource for those new to working in the MRI-linac environment. Those familiar with these subjects may wish to skip straight to the following section on site development, however we draw your attention to section ‘Impact of Static Field on Treatment Beam’, which describes issues novel to the MRI-linac environment that are worth consideration.

The physical origins of magnetic field hazards in the MRI-linac environment and the technical parameters affecting them are similar to those encountered in diagnostic MR, and are described in guidance [1–3] and elsewhere [12, 13]. MRI-linac technology, ancillary equipment, patients, and workflows are sufficiently different to diagnostic MR that hazards need to be understood, reassessed, and safe practices adapted or developed. Safety policies and procedures therefore need careful and specific consideration in the MRI-linac context to best manage risks and benefits for patients and staff. The following sub-sections introduce significant parameters, fields, effects, and hazards that need to be understood and considered when managing MRI-linac safety.

### Limited parameters

All commercially available MRI-linac equipment is designed to meet basic safety and performance standards published by the International Electrotechnical Commission (IEC) [14]. Equipment must comply with specific IEC limited parameters which aim to limit or control potentially harmful effects. Information on the health effects of exposure to magnetic and RF electromagnetic fields associated with the use of MR equipment is also provided by the International Commission on Non-Ionising Radiation Protection (ICNIRP) [15–20].

IEC and ICNIRP limited parameters are often framed in terms of escalating risk:

- Normal operating mode is intended to present minimal risk to the majority of the population
- First level controlled operating mode may cause some physiological distress and a risk versus benefit assessment is required
- Second level controlled operating mode is a research mode that may produce significant risk to patients and can only be used with ethics approval and clinical supervision.

The parameters considered include static field (*B*_*0*_), time-varying gradients (*dB/dt*), specific absorption rate (SAR) by body part, and temperature rise; limit values for each can be found in the IEC and ICNIRP references.

Compliance with limited parameters does not guarantee safety and some knowledge of the physics and physiological interactions is essential for all MRI-linac staff. More in-depth knowledge and experience of complex scanning situations is essential for designated appointees (MRMD, MRSO, MRSE).

### Static field

The commonly used term ‘field strength’ is usually denoted *B*_*0*_ and refers to the z component of the static and nominally uniform magnetic flux density, directed along the bore. This static field polarises tissue magnetisation to give a measurable physiological signal. A field strength of *B*_*0*_ = 1.5 Telsa (T) is approximately 30,000 times the strength of the earth’s magnetic field and to the MR novice can be an entirely unintuitive concept with unexpected and potentially lethal consequences.

As an object approaches the MRI-linac, it first experiences the fringe field which is unique to each installation and extends several metres beyond the magnet bore. The distant fringe field can affect the function of electro-mechanical devices such as conventional linear accelerators which may therefore have to be sited over eight metres away [21], and some MRI scanners may have to be placed over ten metres away. Movement of large ferromagnetic objects in the distant fringe field can affect *B*_*0*_ homogeneity, disrupting image encoding and causing distortion and image artefacts. MRI-linac suppliers will provide guidance on movement of metallic objects in the vicinity of the MRI-linac.

There is little immediate risk until the fringe field reaches a level of 0.5mT, at which point there is a chance that pacemaker function could be affected, and zoning and screening concepts are required to control access. As we get closer to the bore the 3mT field line is another useful demarcation point as there is now a significant risk that the attractive force on ferromagnetic magnetic objects could be greater than the gravitational force, and objects may be accelerated towards the bore or twisted to align with the field. Access to the 0.5 mT field line should be controlled and it may be helpful to demarcate the 3mT field.

Beyond the 3mT field line, lies the area of greatest risk and it is useful to take a closer look at interactions with the magnetic field. Superconducting MR units are usually shielded with a second superconducting coil surrounding the inner imaging coils, this secondary coil generates an opposing field which acts to significantly limit the extent of the fringe field and allow a smaller MR footprint. This shielding also has the effect of greatly increasing field gradients near the entrances to the bore.

Significant risk arises from magneto-mechanical effects on ferromagnetic objects with a high positive magnetic susceptibility, giving a high internal magnetisation up to the saturation limit. It is possible to predict the behaviour of soft ferromagnetic objects with simple geometries [22], and this allows us to make some observations.

The translational force on soft ferromagnetic objects:

- Varies as the product of field strength and field gradient
- Does not depend on susceptibility
- Is strongly non-linear with distance and is maximal near the bore entrance
- Is strongly dependent on shape and alignment
- Can increase to many times the object’s weight over a short distance
- Can result in very high velocities (10’s m/s) if the object is released

The torque and rotational force on soft ferromagnetic objects:

- Varies as the field squared
- Is non-linear with distance and is maximal in the bore
- Does not depend on susceptibility
- Is strongly dependent on shape and alignment
- Rotational force is inversely related to object length
- Implanted ferromagnetic objects present a very high risk

We may illustrate these behaviours with an example (following McRobbie [22]): a 1 kg cylindrical steel socket wrench of length 250 mm and diameter 25 mm, aligned along the central axis and within a metre of the bore entrance of a typical scanner could experience a field of 100 mT and a gradient of 0.6 T/m. Such a wrench would be subject to a translational force of over 70 N, a steel sphere of the same weight at the same position would experience a force of 18 N. If the wrench were rotated by 45° to the bore axis it would experience a torque of over 80 Nm whereas the sphere will experience zero torque.

The behaviour of hard, permanently magnetised object (such as found in cochlear implants) is less predictable. We can however, say that initial translation and rotational forces are likely to exceed those of a soft ferromagnetic object.

This brief discussion on ferromagnetic objects should give some feel for the processes, forces and effects involved. Characterisation of specific devices and prediction of forces can quickly become complex as all ferromagnetic components need to be identified, sometimes requiring detailed information from manufacturers. The interested reader is referred to texts such as McRobbie’s Essentials of MRI safety [22] for further study, which should be viewed as essential for anyone seeking a deeper understanding of the physics of MR safety.

The static field will have the same effects on implants inside the body and can also affect the operation and safety of implanted active devices. Implant manufacturer’s conditions (see Sect. “Understanding and interpretation of manufacturers conditions”) provide guidance on how to safely scan or not scan patients with such implants.

An additional consideration is the speed of conductive implant movement through the fringe field, this can induce eddy currents that act to oppose the motion (Lenz force). Such forces are likely to be small in magnitude but slow patient and table movements may be necessary to avoid pain or harm, depending on type and location of implant. If an unknown implant or foreign object is discovered during scanning the supervising radiation oncologist must be advised and the study managed to minimise risk, including considering slow table movements.

There are other direct static field effects which should be mentioned but which lead to discomfort or erroneous physiological readings rather than any risk to life, these include:

- Acute sensory effects due to movement [18]: metallic taste, vertigo, nausea.
- ECG artefacts [23] due to flowing blood inducing an electric field (magneto-hydrodynamic effect).

### Time-varying gradient fields (*dB/dt*)

Gradient fields are used to both produce MR signal echoes and to spatially encode those signals into the k-space information required to form an image. The most significant physiological effects arising from gradient fields are discomfort or hearing loss from acoustic noise, and peripheral nerve stimulation (PNS) caused by excitation of peripheral nerves by electrical voltage potentials resulting from induced electric fields.

#### Acoustic noise

The dominant noise heard during MR imaging is generated from vibrations in the gradient coils. In simple terms, the MR gradient system can be compared to a large loudspeaker with audio-frequency currents (the gradients) driven through a coil within a magnet. The resulting Sound Pressure Level (SPL) is measured in decibels (dB) with different frequency weightings (A, C, and Z [24]) used according to the application. IEC [14] sets an absolute limit on scanner noise of 140 dB(Z) for the unweighted peak SPL and requires the use of hearing protection if scanner noise exceeds 99 dB(A). In basic terms, temporary hearing damage can occur for brief exposures at 100 dB(A) and permanent hearing damage can occur at 120 dB(A). Generally, a sound pressure level (SPL) greater than 85 dB(A) over a prolonged period (8 h) is considered unsafe [15]. Scanner noise depends on the specific sequence, it is often over 100 dB(A) and can reach levels above 120 dB(A) for some sequences.

RANZCR [1] simply and usefully states that ear protection must be worn by anyone in the scanner room during scan acquisition. Patient communication is important during MR scanning, so manufacturer-supplied headphones often have plastic tubing allowing pneumatic sound transmission, but which compromises protection. Well-fitting, Class 5 [25] ear plugs should therefore always be used in combination with such headphones. Particularly loud sequences may have to be limited in duration, and appropriately sized ear protection may be required for smaller patients.

#### Peripheral nerve stimulation

Sensation of discomfort due to PNS can be subjective and can range from a tingling or tapping sensation to painful muscle contractions. As gradient fields change in magnitude or polarity, electric fields are induced in tissues with a strength dependent on the rate of change of the gradient fields *(dB/dt*) [26]. These electric fields can cause stimulation via depolarisation of peripheral nerves.

Nerve stimulation can be described by a strength duration (SD) curve [14] which models the response based on a minimum stimulation threshold known as the Rheobase (occurring for long duration stimuli) and the Chronaxie, which is the threshold duration for a stimulus of double the Rheobase. SD curves [27, 28] show that for stimulation to occur, a higher rate of change of gradient field (*dB/dt*) is required for shorter gradient rise and fall times. The SD curve can also be formulated to identify and avoid gradient parameter combinations that are likely to cause PNS [22, 29].

Stimulation is more likely for *y*-gradient switching (i.e. the AP direction for a prone or supine patient) due to the large patient area coincident with the high gradient region near the top of the patient couch. PNS may also be more likely for oblique slices where more than one gradient is activated at the same time and for high resolution images using stronger gradients.

IEC [14] and ICNIRP [15] provide PNS guidance with the Normal mode limit derived from 80% of the median perception threshold of a population, and the First Level and Second Level Controlled modes increasing the limit to 100% and 120% respectively. Scanners provide stimulation predictions calculated for each sequence and weighted to account for the direction of the applied gradient. These simulations are based on either the IEC default SD [14] curve or the manufacturer’s own model derived from volunteer exposures.

Both PNS and high acoustic noise levels are more likely for pulse sequences using stronger gradient amplitudes such as diffusion weighted imaging (DWI) or for rapid gradient switching, especially with reversed polarity lobes, such as echo planar imaging (EPI). The highest fields and therefore risk of nerve stimulation may be outside the field of view and around the periphery of the patient, coinciding with the location of many peripheral, myelinated sensory and motor nerves.

An additional but less serious risk comes from fast head movement or shaking at the scanner bore entrance which may, rarely, cause stimulation of the retinal receptors or optic nerve and give rise to brief flashes of perceived light known as magneto-phosphenes. ICNIRP [30] considers the onset of magneto-phosphenes to be the most sensitive acute sensory effect arising from low frequency time varying magnetic fields and sets occupational exposure limits at an avoidance level.

Time-varying gradient fields can also cause vibration, induce currents and result in heating of implanted devices, especially larger devices with high conductivity. The function and safety of active implanted or nearby devices may also be compromised. Implant manufacturer’s conditions (see Sect. “Understanding and interpretation of manufacturers conditions”) provide guidance on how to safely scan or not scan patients with such implants.

### Radiofrequency (RF) field

Transmitted radiofrequency (RF) pulses form the magnetic *B*_*1*_ field, oriented at right angles to the main *B*_*0*_ field. The circularly polarised *B*_*1*_ pulse excites the spins in the selected tissue to nutate or flip from the initial, relaxed state along the bore and induce measurable RF signals in the orthogonal plane. There are two significant mechanisms for harm arising directly from RF pulses: patient heating and tissue burns. These effects are not limited to the portion of the patient directly within the transmit coil, with induced fields potentially extending tens of centimetres beyond [31].

#### Temperature rise

In the process of RF resonance, electric fields are induced in the patient and significant energy can be deposited, leading to potentially harmful temperature rises. The IEC [14] defined Normal Operating Level limits predicted core temperature rise to 0.5 °C and is not expected to cause any physiological stress, whereas the higher operating levels involve increasing likelihood of stress and require higher levels of justification, authority and supervision.

It is not practicable to measure core temperature in every patient, so surrogate limited parameters that predict temperature rise are calculated based on sequence parameters and patient size. Of these surrogates, the specific absorption rate (SAR), defined as the power absorbed per unit mass (W/kg), is the most familiar. SAR depends on multiple parameters and can be formulated for simple geometries [22, 32]. However the only way to accurately predict local SAR is by numerical modelling which is not feasible for every patient but can be calculated by manufacturers and, together with gel-phantom measurements [33], forms the basis of the scanners’ approximately predictive SAR algorithms. With the correct patient weight (and sometimes height) entered, the scanner will supply predicted SAR values for each sequence. During scanning, the current SAR is updated every 10 s, with relevant RF energy absorption factors and limit values displayed for monitoring.

SAR is usually presented in terms of the whole-body, head, or partial-body average, but energy is not uniformly deposited throughout the body and hotspots can occur where local SAR could be an order of magnitude higher than the average, and local temperature rise could be significantly higher [34]. The presence of conductive implants can also induce hotspots, and various cooling mechanisms further complicate the picture.

In the absence of conductive implants and for patients with a normal thermal regulatory function, SAR levels usually provide sufficient guidance to manage temperature rise safely. Patients requiring extra caution include the febrile, neonates and foetuses, and those with compromised thermal regulation due to factors such as: diabetes, cardiovascular disease, some medications, old age, obesity, and pregnancy. Worthy of special mention is the foetus: a local hot spot could significantly raise the foetal temperature and normal cooling mechanisms are not as efficient. A conservative approach is therefore recommended [1]: using quadrature rather than parallel transmission RF, and avoiding First Level Operating Level unless justified by a risk–benefit assessment by the responsible Radiation Oncologist.

There are two more limited parameters that provide additional RF information, useful in specific circumstances [14]: Specific energy dose (SED, J/kg), and *B*_*1*_+_*rms*_ (µT). SED is defined as the RF energy absorbed in tissue per kilogram of mass and is equal to the SAR times the scan duration. SED gives a useful measure of the total energy absorption from multiple sequences over a period of time and can be used to give a crude estimate of core temperature rise. The IEC [35] SED limit is 14.4 J/kg, equivalent to a SAR of 4W/kg for one hour of scanning: compliant systems will disable scanning above this level. *B*_*1*_+_*rms*_ refers to the ‘root mean square’ value of the effective RF field averaged over 10 s and is more directly related to induced electric field in tissue and metallic implants as it is derived directly from the coil transmit voltage which is tuned to each patient. *B*_*1*_+_*rms*_ is a useful metric for implant manufacturers to define the parameters at which an MR Conditional device has been demonstrated to be compliant. For a given patient, RF pulse waveform, and *B*_*0*_: *B*_*1*_+_*rms*_ is proportional to the square root of SAR.

#### RF burns

Radiofrequency burns are the most common injury due to MR imaging. Delfino et al. [36] reported that 59% of all incidents reported to the FDA over a 10-year period were due to RF related thermal injury. RF burns can be caused by induction of currents, either in the patient, in implants, or in nearby conductive components. These induced currents can cause large temperature increases and serious injury, especially when concentrated across a small area of tissue.

Patients may be sedated or have impaired awareness or communication abilities, and burns may be due to heating in the subcutaneous fat where there are no pain sensors. The patient therefore cannot always be relied on to alert the operator to pain and a higher level of care and supervision is required in these circumstances.

There are many different pathways, equipment types, and mechanisms that can lead to RF burns including:

- Patient contact or close proximity to scanner bore leading to high RF coupling.
- Large conduction loops due to patient size or positioning, especially when a small contact area forms the loop (e.g. separated legs with touching feet).
- Inappropriate type or setup of physiological leads and electrodes.
- Conductive external fixation or positioning devices.
- Other potentially conductive materials such as tattoos and transdermal medication patches.

As a consequence, there are several measures required to reduce the risk of patient burns, including:

- There should be no metal (or other conductive material) in contact with the patient, including medication patches and clothing; hospital gowns are recommended for all scanning.
- Use non-conductive pads (e.g. foam) to ensure at least 10 mm separation of the patient from the bore covers, coils, or any conductive leads.
- Any leads must not be looped and must be separated, they should also leave the bore in a straight line close to the central axis.
- It should be explained to the patient that they must not cross their arms or legs to avoid large current loops; use padding between upper legs as necessary.

The likelihood of RF tissue heating, or RF burns can be reduced by lowering SAR. Some scanners have different RF pulse modes available with low and high SAR options. SAR can also be lowered by reducing the number of echoes or slices or by increasing TR, though this can affect contrast for T1 weighted sequences. All RF pulses add to SAR so the benefit of increased contrast from ‘fat sat’ or decreased scan time from ‘spoilers’ needs to be weighed against the risk of elevated SAR.

For MRI-linac, imaging may be continuous during treatment, possibly leading to prolonged RF exposure, and sequences should be designed to keep within SAR limits and any implant conditions. For patients in treatment positions that place anatomy near the bore wall, extra care may be needed to ensure there is no skin contact.

RF fields induce currents which may result in heating around implanted devices, especially for long conductive leads, potentially causing serious injury. The function and safety of active implanted devices may also be compromised, potentially disrupting essential life-support functions. Manufacturer’s conditions (see Sect. “Understanding and interpretation of manufacturers conditions”) provide guidance on how to safely scan patients with such implants. Electronic QA or monitoring equipment function also can be affected by RF interference and correct function should be assessed as well as safety.

Understanding mechanisms and hazards of the MR environment is essential in managing the risks of RF burns, staff and patient education is therefore important. Risk can be reduced by careful selection and appropriate use of MR safe or MR Conditional devices. The multiple factors should be understood and considered on a patient-by-patient basis, including: sequence selection, equipment selection and positioning, patient positioning, thermal insulation, and cooling.

### Impact of static field on treatment beam

The introduction of a large magnetic field to the linear accelerator environment can result in significant changes to dose deposition due to the Lorentz force acting on charged particles liberated from material in the beam path. This is true whether the magnetic field orientation is perpendicular to, or parallel with, the radiation beam axis. The parallel magnetic field produces additional entrance dose due to the focusing of contaminant electrons [37], while the perpendicular field has been shown to reduce build-up depth and skew beam penumbra [8].However, there are other effects of the perpendicular field that are more directly relevant to patient safety: the electron return effect [38] (ERE), the electron streaming effect [39] (ESE) and spiralling contaminant electrons [40, 41] (SCE).

#### Electron return effect (ERE)

The Lorentz force acting on a charged particle moving perpendicularly to a magnetic field causes it to turn back on itself in a perpetual cycle, with a radius of curvature dependent upon its velocity and the magnetic flux density. If the charged particle exits a medium into an air cavity it may turn back and deposit its energy in the exit surface. This not only results in increased dose deposited at the upstream surface, as the electrons “return” to the higher density medium, but also reduced dose deposition at the downstream surface due to the reduction in electron flux across the cavity. If the length of the cavity is greater than the radius of curvature of the electrons and the photon beam axis is normal to the exit surface, then effectively all of the electrons exiting the medium into air will return back to the medium [38]; however, if the beam axis is not normal to the exit surface this may result in streaming electrons (see below).

Higher exit doses are seen on MRI-linac systems with a perpendicular magnetic field when the beam exits into the air rather than a couch or patient positioning system [42]. This can be mitigated by the application of tissue-equivalent bolus material [39, 43], so long as it does not interfere with other treatment beams. Extra care must also be taken to ensure beam exit does not coincide with entry of an opposing beam or the skin-sparing benefit of the megavoltage photon beam may be undermined.

#### Electron streaming effect (ESE)

The electron streaming effect is an alternative outcome to ERE for charged particles exiting material in the beam path in the presence of a perpendicular magnetic field. Furthermore, electrons scattered back out of the entrance surface may also be affected. Virtually all exiting electrons will have a non-zero velocity component parallel to the axis of the magnetic field causing them to not only circle back on themselves but to spiral along the conceptual lines of magnetic field. When exiting a surface that is not parallel to the magnetic field they may continue to drift until they encounter either patient or hardware. Therefore, ESE can result in significant skin dose to the patient well outside the intended treatment area.

**Fig. 3:**
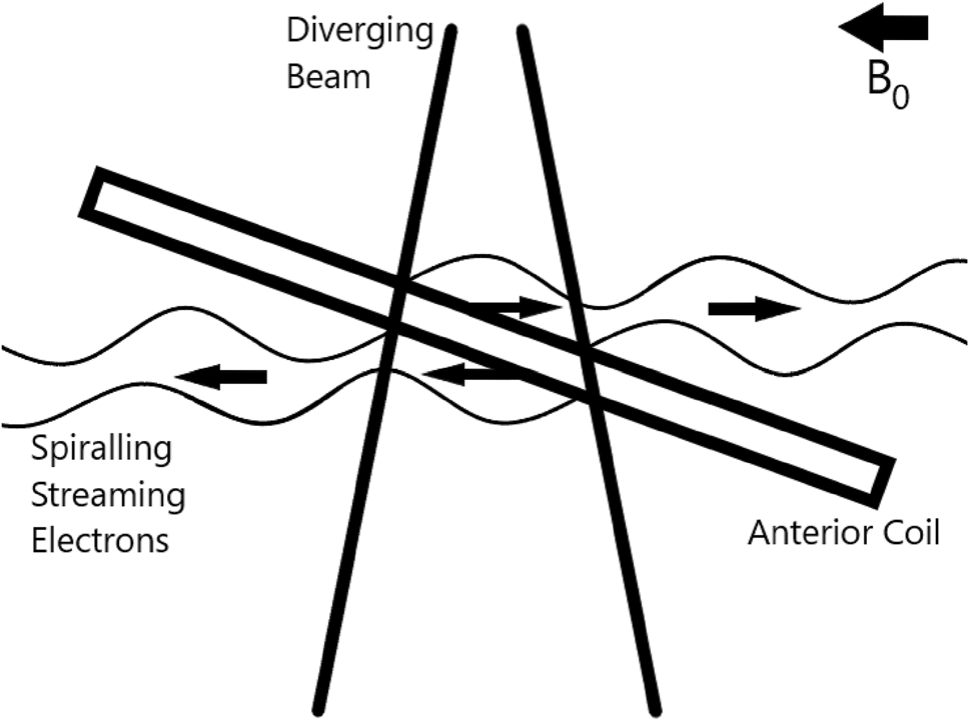
Electrons streaming from exit and entry surfaces from a tilted anterior coil

Baines et al. [44] measured out-of-field ESE dose of up to 28.0% of the maximum in-field dose (D_max_) due to electrons liberated from the anterior coil in a 1.5 T perpendicular magnetic field. By comparison, the ESE dose calculated by the treatment planning system (TPS), was less than measured by up to 13.0%. The same work reported approximately 14 Gy (36% of the prescribed dose) to the surface of bolus protecting the ear over the course of a supraclavicular nodal treatment. Additionally, doses due to ESE from the anterior imaging coil and vacuum immobilisation bag can be clinically significant [45]. The magnitude of the effect at the entrance surface of the anterior coil was comparable to that at the exit surface when the coil was tilted, with the two streams travelling in opposite directions (Fig. 3).

Malkov et al. [39] simulated an exit surface angled at 45 degrees to both the photon beam and magnetic field axes and found up to 39.0% and 35.8% of D_max_ deposited out-of-field in 0.35 T and 1.5 T perpendicular fields, respectively. Dose values dropped below 2% within the first 1 cm of the out-of-field phantom, demonstrating the effectiveness of protective bolus against stray ESE dose.

ESE presents a significant safety concern, particularly as it is novel to both conventional radiation (linac) safety and established MR safety cultures; as such, it will be alien to new users regardless of experience. Careful consideration is needed during treatment simulation, planning, patient setup, and potentially end-to-end QA where irradiation of electronic devices may be of concern. It may be necessary to acquire larger volume planning CT scans to calculate out-of-field dose, and to apply bolus material of at least 10 mm thickness to absorb unwanted dose to the affected area.

#### Spiralling contaminant electrons (SCE)

Spiralling contaminant electrons (SCE) are those that are produced by photon interactions with the air and are then either focused onto the surface by the parallel magnetic field or carried out-of-field by the perpendicular magnetic field [40]. While the effect is not as dramatic as ESE, SCE in the perpendicular magnetic field can produce out-of-field doses of approximately 4%–5% of D_max_ [39]; this is higher than is tolerable on a standard linac and therefore should be properly assessed and accounted for.

## Site design

As with all MR modalities, there are certain essential components that need to be accommodated in suitable facilities [46]. All RANZCR guidance [1] around site layout and management applies to the MRI-linac environment with additional considerations [47–49]. It is recommended that early in the planning stage, a multidisciplinary group is formed with representation of all stakeholders and that strategic guidance and technical expertise are available to guide the project.

Environmental considerations are ongoing; for example, certain areas may need long-term access control and neighbouring facilities should be made aware of the presence of the MRI-linac and the need to inform the MRI-linac owners of any planned changes to buildings, use, or traffic.

### MRI-linac suite requirements

Some MRI-linac components can have significant impact on or be affected by the neighbouring environment. A new-build gives flexibility at the planning stage whereas achieving an ideal MRI-linac layout in an existing building may involve significant expense and challenges or require additional procedural solutions to manage safety concerns.

The MRI-linac bunker itself requires appropriate levels of radiation shielding [50, 51] plus a Faraday or RF cage to encompass the MR components of the modality and provide sufficient attenuation of RF signals to avoid interference. As discussed in Sect. “Static field”, the distant fringe field may disrupt or influence the function of electro-mechanical equipment such as conventional linacs [21]. Estimated fringe fields should be considered during planning and magnetic shielding installed if necessary. The fringe field should be mapped after MRI-linac installation to ensure no unforeseen effects on the environment, use of a three-axis gaussmeter is recommended. In accordance with RANZCR [1], fringe field maps should be kept on record and shown to staff as part of training.

The more local magnetic fringe field is potentially dangerous and the 0.5mT line should, ideally, be completely within the bunker and must be under strict access control [1]. Bunker doors and mazes are often designed around radiation safety rather than MR safety and both factors need to be accommodated when considering layouts and workflows.

### Access restriction and zoning

ACPSEM and RANZCR [1] support the ACR [2]-defined four safety zones within MRI-linac facilities. These designated Zones I through to Zone IV demarcate areas with potential safety concerns and controls corresponding to increasing magnetic field exposure and risk to staff, patients, and visitors. For new constructions, the ACR four safety zone system is considered mandatory [1]. For existing facilities, every effort should be made to designate and implement the four-safety zone system or, if this is not feasible, achieve similar control through other means.

*Zone I* is freely accessible to the public without supervision, this area is usually outside the MRI-linac environment.

*Zone II* acts as the boundary between Zone 1 and the strictly controlled Zones III and IV and is often a patient waiting or reception area. Patients should be supervised in Zone II and it provides an appropriate space for screening, medical history and gowning. It is also recommended that patient preparation and resuscitation occurs in this Zone.

*Zone III* is a strictly controlled area with restricted access, where the presence of unscreened non-MR personnel or ferromagnetic objects can result in serious injury or death. Access should be controlled by adequately trained MR personnel who should supervise patients, visitors, and non-MR-trained staff at all times. MR personnel must have visibility of all non-trained persons, especially around the entrance to Zone IV. Any resuscitation in Zone III would require additional procedures to ensure MR Unsafe objects are safely managed.

*Zone IV* is the room or bunker containing the MRI-linac and must have strictly supervised access with monitoring of the bunker door or maze entry. It should be clearly demarcated and marked with signage as being hazardous due to the presence of very strong magnetic fields. The door should remain shut or a temporary barrier should be used for the brief periods when the door is open.

**Fig. 4:**
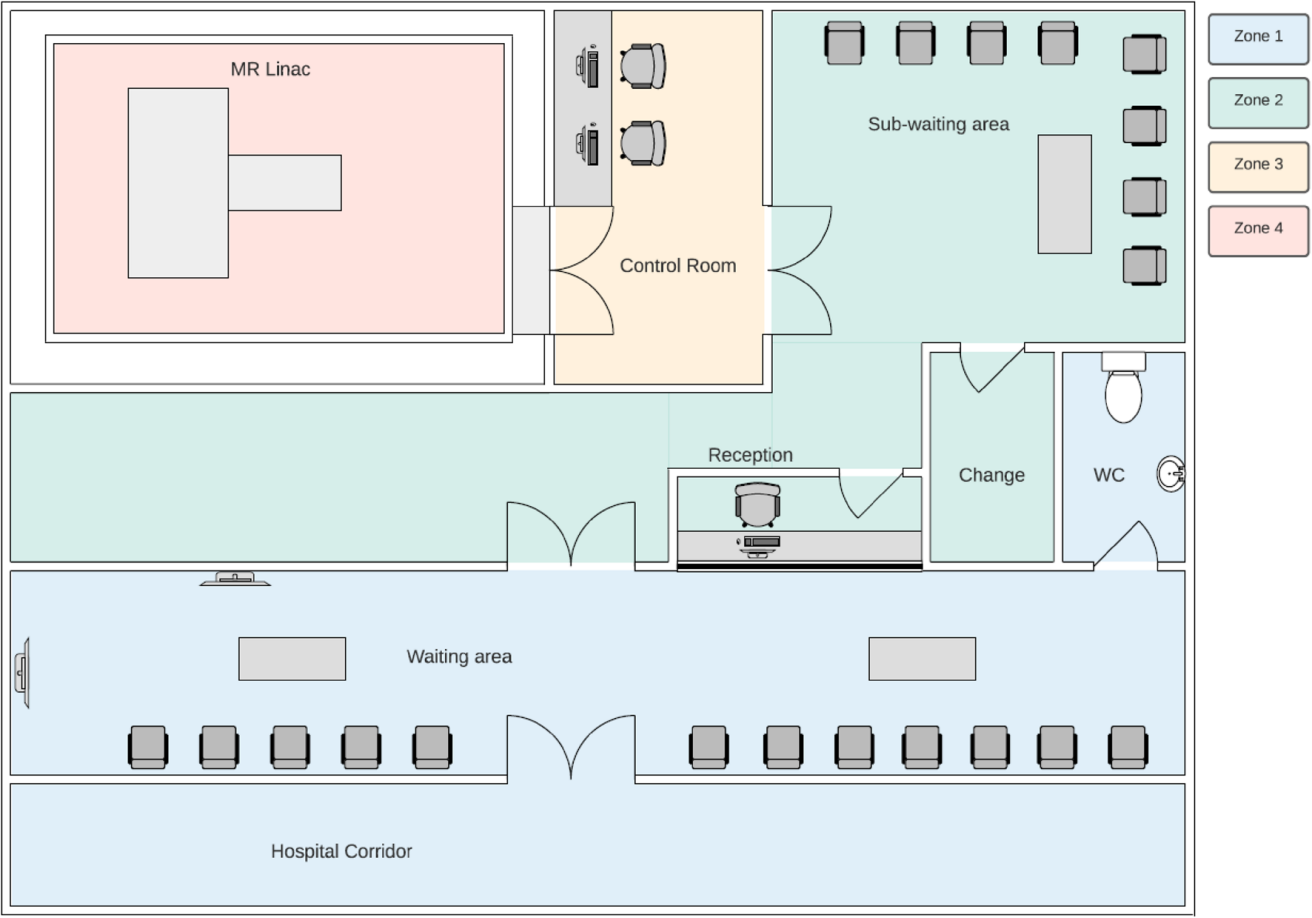
Example layout of MRI-linac suite showing demarcated safety zones

An example MRI-linac suite layout with appropriate zoning is presented in Fig. 4. For some installations it may be challenging to achieve a clear and simple progression of zoning, and additional procedural and security controls may be necessary to capture the intent of the ACR system, including the following:

1. Preventing persons potentially at risk from exposure to magnetic fields from being exposed to fields greater than 0.5 mT. This includes screening to identify those at risk, such as those with implanted electronic cardiac devices (e.g. pacemakers), and controlling access to all potentially hazardous areas.
2. Defining a monitored and restricted access “buffer zone” around the MRI-linac room which is free of potentially hazardous metal objects to minimise the risk of accidental transport of a hazardous object into the scan room. All patients, visitors and non-MR trained staff must be screened for MR hazards before entering this zone.
3. Entry to Zone IV carries the highest risk and various protocols and procedures are useful in reducing inadvertent entry of dangerous objects or at-risk people, including:
  A time-out for a final safety assessment at the entrance to Zone IV
  Use of a plastic link chain or retractable barrier with warning signage to block entry to Zone IV when the door is open
  Hazard signage that is visible whether the door is open or closed.
4. Consideration and planning for emergency situations such as fire, quench, and resuscitation. For example, MR-Safe fire extinguishers must be available, safe evacuation of staff and patients must be ensured, and a resuscitation area in Zone II is recommended to facilitate safe access by non-MR trained emergency services.
5. In some bunker layouts, engineering room access is through the MRI-linac room and it may be necessary (though undesirable) to transport ferromagnetic equipment through part of Zone IV. This is potentially very dangerous and extreme caution and adherence to strict protocols is required. In these circumstances, it is advised to mark the location of the 3mT field line with floor markings and signage as an additional warning of the imminent risk of ferromagnetic projectile hazard.

### Equipment storage, and control

During site design, it is important to consider equipment storage, control, and any transport between MR and non-MR environments. Ideally, any equipment dedicated to the MR environment should be stored nearby in an “MR Equipment” room. If equipment cannot be dedicated to the MR environment, written procedures should be developed for the safe transfer of equipment between environments.

## Magnetic resonance personnel

It is recommended that the MRSE is heavily involved in local safety training, including summarising electro-magnetic theory, effects, and hazards in an understandable way. Development of safe scanning and treatment workflows requires a collaborative approach involving all MRI-linac staff. Safety resources, training material, and procedures should be developed and presented appropriately for the different roles and activities of personnel involved. All safety training should be documented and there should be annual refreshers. Target populations would include all radiotherapy department employees, facility maintenance and cleaning staff, and emergency personnel. The training levels described in RANZCR guidance [1] and below are the minimum required.

### Training status definition

The additional hazards encountered in MRI-linac, especially when combined with an inexperienced workforce, require a special focus on safety training. All staff who regularly work is the same department or location as the MRI-linac are recommended to receive Level 1 training. The training, and supervisory requirements for Level 1 trained personnel should follow those described in RANZCR guidance [1].

#### Level 1 MRI-linac training

- Definitions, rules and procedures for Controlled Areas.
- Use of hearing protection.
- Emergency procedures.
- Quench procedures.
- Magnet safety screening.
- Risks associated with contrast agents and active implants.

The safety of non-MRI personnel who are regular visitors to MRI-linac but have a defined and limited task (for example cleaners or site maintenance staff) may be managed through additional training specific to the person and their role, with a reduced level of supervision. Alternatively, trained MR personnel could take over some duties normally done by others.

Level 2 trained personnel are responsible for their own and others’ safety and the following training topics should be covered which include RANZCR [1] recommendations. ACPSEM recommends that all Medical Physicists in a department which houses a MRI-linac should receive Level 2 training.

#### Level 2 MRI-linac training

- Principles of electrical, static field, gradient field, and RF safety;
- Exposure limits;
- Cryogen hazards
- Implant conditions
- Impact of magnetic field on treatment beam
- Safe use of ancillary equipment
- Assessment of manufacturers conditions
- Managing artefacts, planning, and dosimetry around implants and positioning equipment

#### Level 3 MRI-linac training

- Physics and bio-effects of magnetic fields
- Safety assessment and management of ancillary equipment
- Safety assessment of complex scanning and treatments
- Development of safety procedures to align with guidance and legislation

Those with a leadership role with regard to MR safety (MRMD, MRSO, MRSE) should be thoroughly familiar with all training requirements, including Level 3.

### Staffing levels and working alone

It is strongly recommended that during patient setup, scanning or treatment there is a minimum of two MRI-linac-trained personnel present, with at least one trained to Level 2. One member of staff should deal with patient setup, scanning and treatment, whilst the other is able to monitor Zone III and deal with any distractions. Depending on site layout, video surveillance or other measures may be needed to ensure adequate monitoring of Zones III and IV.

During QA it is recommended that at least two Medical Physicists are present, if this is not always possible, the facility’s lone worker policy should address MRI-linac hazards. In the event of a lone-worker in Zone IV, additional precautions are strongly recommended, such as a plastic chain or retractable webbing barrier across the open entry to the scan room with an additional warning notice.

## Screening of patients and others

RANZCR [1] patient screening recommendations should be followed, these should be adapted to the local patient population, planning, and treatment workflows.

There should be a three-stage screening process as described in RANZCR [1] guidelines. The latter two screenings should be conducted by MRI-linac-trained personnel, one of whom should sign or initial the form and take responsibility for entering it into the patient’s record.

### Referral pre-screening

- The referrer should use a specific MRI-linac request form to confirm no contraindication to MRI-linac, note the make and model of any implants, and specify the particular metals present in any implants near the treatment site.

Subsequent screenings should not solely rely on information provided in the referral pre-screening form which acts only as an early flag for some issues. The second screening should be thorough and accurate and reassess all aspects of patient safety as set out in screening procedures. Any novel or complex screening findings should be referred to the MRSO or MRSE as necessary. The third, review screening should be conducted by the RT performing the examination.

All other persons entering Zone III should be screened prior to entering, this may include carers accompanying patients, maintenance staff, emergency personnel, and others. The screening should be tailored to the person, consider their clothing, and include questions aimed at uncovering hidden hazards in the specific circumstances. Consideration should be given to asking those being screened to perform a self-pat-down to check for missed or forgotten items.

## MR safety classification

All devices, implants, and equipment should be labelled following RANZCR guidelines and the recommendations of ASTM Standard F2503 [52]. This standard defines three categories as follows:

- MR Safe: an item that poses no known hazards resulting from exposure to any MR environment. MR Safe items are composed of materials that are electrically nonconductive, non-metallic, and nonmagnetic.
- MR conditional: an item with demonstrated safety in the MR environment within defined conditions. At a minimum, conditions address the static magnetic field, the switched gradient magnetic field and the radiofrequency fields. Additional conditions, including specific configurations of the item, may be required.
- MR Unsafe: an item which poses unacceptable risks to the patient, medical staff or other persons within the MR environment.

**Fig. 5:**
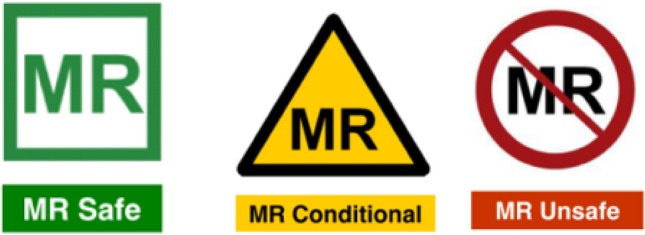
ASTM [52] Standard coloured icons for MR compatibility labelling

Coloured MR icons are also defined as shown in Fig. 5

### Verification of status

RANZCR guidance notes that the only reliable sources of information are the manufacturer’s instructions for use, a credible testing authority, or peer-reviewed literature. At a first MRI-linac installation, there may be essential ancillary equipment with no such literature available. In these circumstances the MRSO or MRSE should be consulted, and a thorough risk assessment performed and documented. If components and hazards cannot be positively identified the device should be categorised as MR unsafe.

## Managing ancillary MRI-linac equipment

The management of safety around radiotherapy-specific ancillary equipment needs special care due to frequent use and a wide range including: trolleys, patient immobilisation equipment, brachytherapy applicators, flat table inserts, RF coil supports, and QA equipment. This section refers to ancillary and QA equipment only, it does not cover patient monitoring equipment or any implanted devices which are considered separately in the next section.

It is recommended that a written protocol should be developed to guide safe ancillary equipment management, including the following considerations:

- Appropriate storage, transport, and transfer.
- Controlled equipment register.
- Procedures for safety assessments and labelling.
- Safe use including any limitations or concerns.

Immobilisation and positioning devices are crucial for accurate and consistent patient positioning from simulation to treatment and are most often supplied by registered medical device manufacturers. Manufacturer’s MR conditions should be strictly followed for these devices where this information is supplied. If no manufacturer’s MR safety information is supplied and for in-house manufactured equipment, a documented QA process is recommended to assess safety, including: electrical conductivity, ferromagnetic properties, imaging artefacts, dose attenuation, and physical compatibility with coils [53].

Phantoms are an essential part of the quality assurance processes used in commissioning and monitoring the performance of an MRI-linac Unit. These can be supplied directly from medical device manufacturers, but often require the use of existing in-house chambers, cabling and various inserts. Existing phantoms and in-house jigs should be assessed for MR safety and labelled accordingly. It may be possible to replace some equipment components with in-house manufactured parts: non-conductive, low susceptibility, plastic materials are preferred [54, 55] and 3D printing may be a useful solution. It is also worth mentioning that cabling for QA equipment may affect image quality and it is therefore recommended that ancillary cabling is removed, or any effects evaluated during image quality tests.

Linac-specific QA devices, including dosimetry equipment, often contain active components. Consideration must be given to the following:

- Manufacturer guidelines specific to the use of device in the MRI-linac; note any field strength and gradient conditions, lead placements and configurations, and device orientation to bore.
- Electronic QA or monitoring equipment function can be affected by fringe fields as low as 0.1mT; correct function and safety should be assessed and recorded.

Carbon-fibre is a conductive material commonly used in radiotherapy which can be associated with RF issues such as heating, burns, or RF shielding, depending on the configuration and placement of the device [56]. Compared to metals, carbon fibre may have the advantage of reduced image artefacts and treatment beam attenuation [57] but safety concerns have to be well understood and carefully assessed. Devices with electrically conductive components may also be affected by currents induced by movement through a static magnetic field (see Table 2 below for more detail).

**Table 2:** Categorisation of interactions of passive devices with magnetic fields in MR (modified from McRobbie [22])^*^

Effect on the Device	Interaction		
	Static magnetic forces	Magnetic forces due to motion	Induction
Translation	Static field *B*_*0*_ Spatial gradient *dB/dz*	Lenz’s Law: Static field gradient *dB/dz* velocity *v*	–
Torque (twisting)	Static field *B*_*0*_	Lenz’s Law: Static field gradient *dB/dz* velocity *v*	–
Vibration	–	–	Gradient *dB/dt*
Electric currents	–	Static field gradient *dB/dz *Velocity *v*	Gradient *dB/dt *RF *dB/dt* (frequency, *B*_*1*_)
Localized heating	–	–	Gradient dB/dt Duty cycle; RF dB/dt Frequency Amplitude Duty cycle

^*^Republished with permission of John Wiley & Sons, from essentials of MRI safety, Donald McRobbie, 1st Ed, 2020; permission conveyed through Copyright Clearance Center, Inc

It is recommended that anything beyond the simplest safety assessments should be conducted by the MRSE or suitably trained MRSO personnel. Tests themselves may be potentially dangerous and should follow a written protocol and be approached with caution. Tests should be limited to the range of likely scenarios, for example if a QA device never needs to enter the bore or be exposed to RF or gradient fields then these scenarios should not be tested and any documented conditions should state such limits on use.

Risks arise from each of the three magnetic field components that are used for imaging: the *B*_*0*_ static field, the RF transmission field *B*_*1*_, and the time-varying imaging gradients, as discussed in Sect. “MRI-linac parameters, fields, effects and hazards” and summarised in Table 2 for passive devices. Potentially harmful effects may result from contributions from more than one component and as such, specific interactions between these fields and materials need to be considered when assessing the safety of equipment. Active electronic or electro-mechanical devices are also subject to these interactions plus additional and unpredictable consequences to function and performance.

### In-house equipment testing

It is important that any equipment that might be taken into the MRI-linac environment undergoes a safety screening process, each device should be assessed independently for individual MR systems.

A review of manufacturer’s documentation is essential to yield information regarding MR safety and component materials. If there is no manufacturer’s MR safety rating, a good first step in assessing any device should be to contact the supplier and obtain details of the materials used in its construction and of any MR compatibility, or electromagnetic interference data. If no manufacturer MR safety information is available, some in-house assessment may be appropriate though there is limited guidance available on how this might be achieved [58, 59]. Suggested tests include, but are not limited to:

- Check for presence of metallic components by scanning on a CT scanner.
- Check for presence of ferromagnetic components by screening with the use of a hand-held magnet with a flux density of at least 0.2 T.
- If any significant (by length or proportional weight) ferromagnetic or conductive part is identified, a more thorough investigation is recommended.
- Translational force and torque may be assessed following ASTM-2052 2015 [60] and ASTM-2213 2017 [61] Standards if it is safe to do so.
- Identification of any non-metallic but conductive parts: particular attention should be paid to carbon fibre and any conductive polymers.
- In the case of devices with ferromagnetic or conductive moving components, consideration must also be given to the fringe field and any effects on the function of both the equipment and the scanner. An assessment of such effects can be performed, and safe operating distances defined and associated with the equipment in the register.
- In-house assessment of MR Conditional equipment should clearly explain the conditions used for testing which may include the following
  - magnitude and location of maximum magnetic fringe field
  - magnitude and location of the maximum spatial gradient
  - maximum rate of change of the gradient fields (slew rate)
  - RF fields tolerated in terms of RF power deposition and transmit mode (e.g. quadrature or parallel)
- It cannot be assumed that if a device is MR Conditional for a particular magnet strength that this will apply for higher or lower magnetic field strengths.

### Comment on ASTM conditional testing

ASTM has produced Standards [33, 60, 61] to assist with safety assessments of implants and devices in the MR environment; a fourth standard specifies means for assessing image artefacts [62].

- F2052-15 standard test method for measurement of magnetically induced displacement force on medical devices in the magnetic resonance environment
- F2213-17 standard test method for measurement of magnetically induced torque on medical devices in the magnetic resonance environment
- F2182-11a standard test method for measurement of radio frequency induced heating on or near passive implants during magnetic resonance imaging
- F2119-07 standard test method for evaluation of mr image artefacts from passive implants

To fully implement these standards would require specialist equipment plus extensive training and experience. There are however, some tests that may be useful for limited in-house testing of particular hazards such as displacement force or torque. It is important that any such assessment is carried out by appropriately qualified personnel such as a MRSE and that the limits of testing are understood and communicated. These standards do not address the functionality and safety of active devices, which is an especially challenging task and is the responsibility of the device manufacturer.

### Further ancillary equipment safety considerations

- MR Unsafe equipment is not permitted to enter the MRI-linac room: unlabelled equipment must be assumed to be MR Unsafe unless it can be positively and confidently identified as presenting no danger (e.g. a plastic ruler).
- Any new or custom-built equipment should be manufactured with MR safety in mind, including minimising metallic (especially ferromagnetic) and conductive content, and using low susceptibility materials.
- If conditions specify that equipment can only be used in a certain field or field gradient, or at a certain distance from the bore entrance then floor markings or equipment tethering should be considered.
- Electronic QA equipment may not have MR Conditional information available and functionality should be assessed [58, 59]. User expertise and experience, with in-house functional testing to compare outputs under different conditions may give some confidence in safety and functionality.

#### Effect of ancillary equipment on image quality

- Any equipment should, ideally not compromise image quality: for example, dosimetric cables may affect image quality in which case they should be removed.
- Any external laser positioning systems should function appropriately in the magnetic fringe field and not cause RF interference.
- Lasers should be turned off during MR imaging
- Coil placements should minimise distance to the patient and not deform anatomy.

#### Patient safety and ancillary equipment

- Attention should be made to the thermal insulation properties of vacuum cushions and avoidance of any artefact arising from small metal components prior to routine clinical use on the MR systems.
- Hearing protection and patient communication may be restricted by immobilisation devices. A patient hand-held alarm switch should be provided to signal discomfort or distress.
- MR Safe couches or wheelchairs should be provided for patient transfer
- Immobilisation equipment may restrict patient evacuation in the case of emergency. Emergency training should be conducted with this in mind.
- Coils, coil leads, and coil bridges should allow a range of positions and configurations to cope with treatment positions and positioning equipment.

### Ancillary equipment management and control

A controlled equipment register should be established by the MRSE to uniquely identify equipment intended for use in the MRI-linac environment. The register should contain at least the following information for each piece of equipment:

- Unique identifier or asset number.
- Description of construction materials.
- Details of any manufacturer MR safety information with the MR safety status (safe, conditional, unsafe) and full details of any conditions.
- Appropriately detailed description of any in-house testing and resulting conditions.
- Requirements for safe storage, handling, use, and any transfer to and from MR environment.

The Register may also contain checklists of equipment to be used with a time-out at the entry to Zone IV as a further check. All equipment’s safety status should be reassessed following any modification to the equipment, change to the MRI-linac operating parameters, or installation of new MRI-linac equipment.

It is recommended that equipment dedicated to the MRI-linac environment is labelled accordingly and is stored nearby in an “MR Equipment” room. If equipment cannot be dedicated to the MR environment it should be clearly labelled and written procedures developed for the safe transfer of equipment between environments.

In exceptional circumstances, it may be necessary to introduce an unknown or known MR Unsafe device, such as a critical piece of equipment containing significant ferromagnetic components. In these circumstances, the MRSE should be consulted, hazards assessed, and a documented risk assessment and work plan developed. Considerations may include: shape of device, mass of device and of ferromagnetic components if known, fringe field at planned device location and path, and any physical restraints that are considered necessary. All such equipment should be clearly labelled, and the procedure should be directly supervised by the MRSO or MRSE.

## Management of implants and Foreign bodies

RANZCR [1] recommendations on implant and foreign body management should be followed. Gach [63] reports that over 70% of MRI Simulation patients had metal inside their body with 40% having metal in the imaging field of view. Identification of specific implants and foreign body materials is essential to identify and quantify hazards, facilitate any clinical assessment of risk and benefit, and for correct treatment planning.

### Understanding and interpretation of manufacturers conditions

RANZCR guidance [1] recommends that all MR scanning involving implants or devices conforms to the implant manufacturer’s conditions. Any implant should be positively identified and the latest MRI safety information that applies in the relevant local jurisdiction should be sought and followed.

MR conditions state the values of various MR parameters applied during testing or modelling to demonstrate safety, plus other patient and scan conditions. The many varied and interacting mechanisms involved should be well understood to allow a well-informed assessment, the parameters may include:

- Static magnetic field, *B*_*0*_ (T)
- Spatial gradient field, *dB/dz* (T/m)
- Spatial gradient field product, *B*_*0*_⋅*dB/dz* (T^2^/m)
- Gradient slew rate (per axis or effective), (T/m/s)
- SAR, Specific Absorption Rate (whole-body, partial-body, or head), (W/kg)
- *B*_*1*_+_*rms*_ (measure of effective RF field), (µT)
- Choice of transmit coil and RF transmit mode
- Distance of device from scanned volume (m)
- Post-surgery time limits
- Guidance on scanning and rest periods
- Configuration and placement of devices, leads, or electrodes
- Presence or location of other devices, leads, or electrodes
- Patient monitoring and rescue requirements
- Appropriate implant function and parameters within specified range
- Correct implant operation tested before and after scanning

It is important to note that safety has been demonstrated only for the specified conditions and safety cannot be assumed for different parameter values. Conditions may state that safety has been demonstrated for parameters less than or equal to the MR Conditions value, if this is not stated it cannot be assumed. For example, an active implant (function usually dependent on electrical power) may have the condition that is it safe to use in a 3 T static magnetic field, it cannot be assumed that this device would be safe in a 1.5 T field as the different RF frequency could affect function or cause harm by other means.

Correct interpretation of manufacturer’s conditions relies on gathering accurate scanner information from the MRI-linac manufacturer’s specifications, understanding these specifications and applying them to the specific device, patient, and scanning situation. For example, it may be necessary to quantify the maximum spatial gradient field that a synthetic implanted heart valve is likely to encounter during a head scan: the location of the heart valve would have to be related to the manufacturer supplied contour maps or data tables detailing the scanner spatial gradient field, and then compared to the device conditions. This is not a simple task and education, training, and guidance are required. Furthermore, sequences may require modification in order to meet the conditions, such modification requires expertise and may not be allowed.

Implanted radiopaque fiducial markers, made from various materials including metals, are often used for target localisation and motion tracking. These are unlikely to cause displacement or heating issues due to their small size [64] but can create significant image artefact, and true and apparent positions may differ [65].

Elongated conductors such as abandoned leads or carbon fibre rods can act as an efficient antenna and concentrate RF heating at the ends, this is particularly the case for lengths close to the half-wavelength in tissue. For 1.5 T the half wavelength is 20 to 30 cm, and at 3 T it is 10 to 15 cm.

### Off-label scanning

Scanning outside the conditions, also known as non-conditional or off-label scanning has been performed for some specific device types in the diagnostic MRI environment when there is a high level of evidence, expertise and support In the MagnaSafe trial [66] of cardiovascular implantable electronic devices (CIEDs), there was evidence of significant lead impedance changes for some patients, implying possible tissue damage from RF heating.

Off-label scanning of CIEDs should not be attempted at recent installations with inexperienced staff. If it is considered, it must be approached with considerable caution and with multi-disciplinary expert support from Cardiology, Radiology and Medical Physics. RANZCR [1] guidance must be followed, which currently refers to the 2017 heart rhythm society (HRS) expert consensus statement [67], including:

- Scanning in accordance with a written policy approved by the MR medical director
- Following a patient-specific risk–benefit assessment by a Level 2 trained radiologist or radiation oncologist, and with informed consent
- Only consider scanning with expert technical support and physician presence, appropriate physiological monitoring, and emergency life-support immediately available.

Implant manufacturer’s conditions often specify static fields of 1.5 T and 3 T only, any other field strength is outside these conditions. Users of MRI-linac equipment that does not use one of these field strengths would therefore scan such devices off-label [63] and appropriate protocols are essential including monitoring and life-support. Off-label scanning of other non-CIED active devices such as implantable pulse generators (e.g., neurostimulators) is forbidden [63].

### Implants in MRI-linac radiotherapy

Relevant tests considering the dosimetric impact of any implant or foreign body on the patient should be conducted by a qualified medical physicist in line with departmental and international guidelines [68]^.^ The effect of radiation treatment on the functionality of active implants should also be considered [69].

## Entry to MRI-linac bunker

RANZCR guidance [1] and applicable radiotherapy legislation must be followed with appropriate radiation safety considerations such as door interlocks, last man out buttons, interlocked gates, signage, and written procedures to form a robust protocol for bunker entry.

### Removal of ferromagnetic objects

In addition to screening, a hand-held magnet with a flux density of at least 0.2 T can be useful in identifying metallic objects as ferromagnetic or not. Wall-mounted ferromagnetic detectors may be useful but should not be relied on as they can give a false sense of security [70]. Hand-held ferromagnetic and non-ferromagnetic detectors are recommended to check for small, hidden metallic items. Any metal detectors should not be relied on but should supplement a thorough screening process. In all cases, the patient, their clothing, the trolley and any ancillary equipment should be checked before entry.

### Timeout at entry to zone IV

At the entry to Zone IV, it is recommended to consider a timeout procedure immediately before entry to Zone IV to check that the patient, equipment, and any ancillary staff are safe.

## Patient management

RANZCR guidelines [1] on patient management should be followed.

### Modification of scan parameters

All imaging sequences should undergo a thorough, documented commissioning process [71] by the medical physics team to assess image quality, artefact, and distortion implications before being used clinically. Vendor sequences used for treatment should not be modified unless specifically agreed to by the vendor, simulation sequences should be referred to the vendor and may be modified only with a full QA assessment and under the direction of the MR Medical Director.

### Claustrophobia and anxiety

RANZCR guidelines [1] should be followed. A significant proportion of patients undergoing MR examination experience high levels of anxiety caused by the combination of confinement within the bore and the loud noises emitted during scanning. These reactions may be exacerbated by the long in-line planning and treatment times typical for MRI-linac.

### Anaesthesia, sedation and monitoring

RANZCR guidelines [1] should be followed. Patient monitoring and sedation in the MRI-linac environment is challenging and will require extra vigilance and safety precautions due to the lack of an observation window and longer patient extraction times. Waveguides or RF filter plate connections are needed for remote monitoring, if these are not available it may be possible to view monitoring equipment in the bunker using CCTV; any in-bunker equipment must be MR safe or appropriately conditional. The patient must be observable via CCTV at all times, with audio communications, and a hand-held patient-activated alarm. Any patient sedation or monitoring would require a written protocol, including practice emergency extractions, a thorough safety assessment by the MRSE, and a risk–benefit analysis by the MRMD.

### Unanticipated artefacts

Artefacts due to an unsuspected metallic foreign body may have a bigger impact on radiotherapy planning and treatment than for diagnostic scans. Careful consideration of the artefact’s location and distortion impact is needed which may require cessation of the immediate scan and slow removal of the patient from the bore. RANZCR guidelines [1] should be followed and a written protocol developed. Artefacts may also be indicative of a potentially hazardous malfunctioning such as coil coupling.

### Positioning equipment

Care must be taken to ensure that any positioning equipment, boards, or pads do not cause artefacts, affect RF coupling, or affect the treatment beam in any way. Such equipment may not be visible on MR images: if equipment could interfere with treatment, it may be prudent to attach MR markers such as Vitamin D [72] capsules to track position.

Positioning equipment may also reduce airflow and lead to increased patient heating, equipment choice should be appropriate and patient comfort should be monitored.

## Contrast agents

RANZCR guidance [1] should be followed.

### Gadolinium safety in radiotherapy

Recent studies have examined the safety of gadolinium-based contrast agents (GBCA) when combined with radiotherapy. For example, one study investigated the possibility of accelerated gadolinium accumulation in the brain due to radiation damage to the blood–brain barrier [73]. Another group was able to rule out acute kidney injury due to liberation of gadolinium from its chelator under irradiation [74, 75].

It is likely that pre-treatment administration of GBCA on an MRI-linac could improve visualization of critical structures during online plan adaption for some treatment sites; however, before this approach is adopted further research is required to understand the effect of radiation on individual agents, particularly given the frequency of repeat scans and short time interval between MR imaging and irradiation. Research in this area is ongoing and should be monitored closely.

### Potential for gadolinium radiosensitisation

Studies have been conducted into the radiation enhancing effect of high-Z elements within the irradiated volume via auger cascade following the ejection of a K-shell electron [76, 77]. Gadolinium-loaded nanoparticles (GdNP) have been demonstrated to enhance the effect of a monochromatic beam tuned to the energy of the K-shell absorption edge of gadolinium (50.2 keV) [78]. The nanometre range of these auger electrons suggest the gadolinium atoms must be within the cell, preferably close to the nucleus, to be effective. Another study showed increased effectiveness after most of the agent had been washed out, but where the remaining particles were concentrated in certain areas within the tumour [79]. The authors proposed that those GdNP that were not washed out may have adhered to, or been internalised within, the cancerous cells.

The effectiveness of these radiosensitising agents under clinical beam energies is showing mixed results [80–82]. However, given the potential for GBCA to effect radiotherapy dosimetry, further investigation is warranted when significant gadolinium uptake is likely to be present in-field during treatment.

## Noise protection

Ear protection is mandated [1] for all MRI scans. Patient positioning devices such as masks may not allow the wearing of bulky ear protection or may reduce its effectiveness. For treatment sites like brain and head & neck, headphones should not be used during the simulation and treatment process to minimize dosimetric uncertainty in dose estimation and delivery. In which case appropriately fitted ear-plugs with a high noise reduction (Class 5 [25]) should be used.

## Special patient groups

### Patients and volunteers participating in research

RANZCR guidance [1] should be followed with the following amendments.

- Where additional imaging is being carried out during a radiotherapy treatment session, a limit should be set for the maximum overall time the patient/volunteer is on the MRI-linac
- Pregnancy must be excluded for studies involving radiation treatment

### Pregnancy

RANZCR guidance [1] should be followed for imaging-only use of the MRI-linac, including a risk–benefit analysis by the RO and a formal, written informed consent process. In all other circumstances ARPANSA RPS 14.3 [83] applies in Australia and ORS C3 [84] in New Zealand, in addition to RANZCR guidelines.

When a pregnant patient is examined, scanner-specific protocols should be followed to minimise sequence duration, SAR, and noise. Scanning should also be limited to normal mode, use of 1st Level controlled mode would require a risk and benefit analysis, agreement from the referring physician, and supervision by the radiation oncologist.

## Data Availability

Not applicable.

## Appendix: Safety system review and audit

Records of policy reviews and audits must be reviewed at least annually by the MRMD and MR safety committee. Reviews and audits require the close involvement of the MRSE. MRI-linac safety review and audits should consider the following items:

### Facilities

It is crucial that facility layout is carefully planned, hazards identified, and comprehensive strategies implemented to manage risks. Critical components should be regularly reviewed and inspected. Issues include but are not limited to:

- Strategic management, identification of stakeholders, delegation of responsibilities to appointed positions, establishment of MR safety committee
- Bunker and door/maze design, technical room and access, MRI-linac environment and neighbouring facilities
- Commissioning tests of RF Faraday cage, checks of door function and seals, security and monitoring of Zone IV access
- Vibration and fringe field surveys, effects from or on other equipment, access restriction above 0.5 mT
- Layout and access to MR Zones: control, observation, supervision, screening, signage, prep/resus area
- Number of entrances, door function and position, alarms, locks and interlocks.
- Zone IV: oxygen monitor, emergency cryogen exhaust fan, pressure equalisation/escape hatch, signage, temporary door barrier, consider metal detector, cardiac arrest alarm, handheld magnet
- Patient alarm, manual couch release, emergency stop, quench button, Zone IV patient CCTV
- Location and condition of quench pipe, helium transport and storage, warning signs, restricted access
- Ancillary equipment storage, transport and management
- Wave guides and filter plate for monitoring and QA cabling

### Policies, procedures, and documentation

Comprehensive, documented policies and procedures must be developed to identify and manage all MR safety risks to staff, patients, and visitor. Measures include but are not limited to:

- Patient-screening questionnaire, verified removal of unsafe and non-conditional items, timeout at Zone IV door
- Regular checks of safety features functionality including Zone IV door, CCTV, patient alarm and intercom
- MRI-linac safety training, emergency practice drills, MRI-linac fire safety response
- Assessment of impact of MRI-linac (and quench) on nearby devices including linear-accelerators
- Ancillary equipment-register, equipment safety assessment, labelling, and safe use
- Appropriate Zone signage and temporary Zone IV barriers
- Policies on GBCAs, noise protection, pregnancy
- Assessment of implant and ancillary equipment conditions
- Relationships with external staff and organisations
- Records of training, screening, scans, planning, treatment
- Incident reporting and review

## Abbreviations

AAPM: American association of physicists in medicine
ACPSEM: Australasian college of physical scientists and engineers in medicine
ACR: American college of radiology
ARPANSA: Australian radiation protection and nuclear safety agency
ASMIRT: Australian society of medical imaging and radiation therapy
ASTM: American society for testing and materials
CBCT: Cone beam computed tomography
CCTV: Closed circuit television
CIED: Cardiovascular implantable electronic devices
DIMP: Diagnostic imaging medical physicist
ERE: Electron return effect
ESE: Electron streaming effect
GBCA: Gadolinium based contrast agent
HRS: Heart rhythm society
ICNIRP: International commission on non-ionizing radiation protection
IEC: International electrotechnical commission
IPEM: Institute of physics and engineering in medicine
Linac: Linear accelerator
MHRA: Medicines and healthcare products regulatory agency
MRI-linac: Magnetic resonance linear accelerator
MRI-Sim: Magnetic resonance imaging used primarily for planning radiotherapy
MR: Magnetic resonance
MRMD: MR medical director
MRSO: MR safety officer
MRSE: MR safety expert
NZIMRT: New Zealand institute of medical radiation technology
ORS: Office of radiation safety (NZ)
PNS: Peripheral nerve stimulation
RT: Radiation therapy/therapist
RO: Radiation oncologist
ROMP: Radiation oncology medical physicist
SCE: Spiralling contaminant electrons
SAR: Specific absorption rate
SED: Specific energy dose
SID: Source image distance
TPS: Treatment planning system
